# Mechanisms of vapor‐phase antibacterial action of essential oil from Cinnamomum camphora var. *linaloofera Fujita* against *Escherichia coli*

**DOI:** 10.1002/fsn3.1104

**Published:** 2019-07-04

**Authors:** Kegang Wu, Yahui Lin, Xianghua Chai, Xuejuan Duan, Xinxin Zhao, Chen Chun

**Affiliations:** ^1^ College of Chemical Engineering and Light Industry Guangdong University of Technology Guangzhou China; ^2^ Guangdong Provincial Bioengineering Institute (Guangzhou Sugarcane Industry Research Institute) Guangzhou China; ^3^ School of Food Science and Technology Henan University of Technology Zhengzhou China; ^4^ School of Food Science and Engineering South China University of Technology Guangzhou China

**Keywords:** antibacterial mechanism, essential oil, linaloofera fujita, vapor‐phase antimicrobial activity

## Abstract

The purpose of this study was to investigate antibacterial activity of essential oil from Cinnamomum camphora var. linaloofera Fujita (EOL) at vapor phase and its mechanism of bactericidal action against *Escherichia coli*. Results showed that the vapor‐phase EOL had significant antibacterial activity with a minimum inhibitory concentration (MIC) and minimum bactericidal concentration (MBC) of 200 μl/L. Further analyses showed that treatment of *E. coli* with vapor‐phase EOL resulted in partial degradation of cell membrane, increased membrane permeability, leakage of cytoplasm materials, and prominent distortion and shrinkage of bacterial cells. FTIR showed that EOL altered bacterial protein secondary and tertiary structures. GC/MS analysis showed that the components of vapor‐phase EOL included linalool (69.94%), camphor (10.90%), nerolidol (10.92%), and safrole (8.24%), of which linalool had bactericidal activity. Quantum chemical analysis suggested that the antibacterial reactive center of linalool was oxygen atom (O_10_) which transferred electrons during antibacterial action by the donation of electrons.

## 1 INTRODUCTION

The vapor‐phase antibacterial activity of essential oils has broad applications in the field of food preservation, agricultural products preservation, air disinfection, and mildew proofing. Therefore, many studies have investigated the ingredients and antibacterial mechanisms of the essential oils (Chen et al., 2018; Doran, Morden, Dunn, & Edwards‐Jones, 2009; Fisher & Phillips, 2006; Paul, Dubey, Maheswari, & Kang, 2011; Tullio et al., 2007). Studies have shown that antibacterial activity of the essential oil at vapor phase is achieved by multiple mechanisms including cell wall degradation, cell membrane damage, membrane protein structural changes, cytolymph leakage, cytoplasm condensation, and alteration of nuclear activity (Bouhdid et al., 2010; Burt, 2004; Devi, Nisha, Sakthivel, & Pandian, 2010; Tyagi & Malik, 2010; Wang et al., 2019; Zhang et al., 2019). However, the active center of antibacterial substance, the site, and mode of action on bacteria have not been reported about essential oils. Essential oil from Cinnamomum camphora var. linaloofera Fujita (EOL) is isolated from branches and leaves of Cinnamomum, and its main ingredient is linalool (Chen, You, Abbasi, Fu, & Liu, 2015; Liu et al., 2006; Pragadheesh et al., 2013; Singh, Srivastava, Kumar, & Dubey, 2008; Zhang et al., 2019). It has been shown that linalool has antimicrobial effects on the common bacteria and respiratory pathogens in air (Cox et al., 2000; Sato, Krist, & Buchbauer, 2007; Wang et al., 2019; Yamaguchi, Inouye, & Takizawa, 2001). However, the vapor‐phase antibacterial activity and its mechanism of EOL have not been studied.* Escherichia coli* is the most famous bacterium in human and animal intestine and the most widely and deeply studied bacterium in modern biology, the conclusion from *E. coli* by the methods of molecular biology can be used for the study of other creatures, so it is often used widely in scientific research as model microorganism. In this study, we elucidated the vapor‐phase antibacterial mechanisms of EOL by investigating microscopic structure, membrane permeability, and biological macromolecular structural changes of *E. coli*. We also identified the antibacterial active center of the vapor‐phase EOL by quantum chemical analysis.

## 2 MATERIALS AND METHODS

### 2.1 Experimental materials

Essential oils including linaloe wood oil, litsea cubeba oil, clove oil, cassia oil, star‐anise oil, rosemary oil, mentha arvensis oil, eucalyptus oil, tea tree oil, orange oil, marjoram oil, and clary sage oil were provided by Guangzhou Baihua Spice Co., Ltd. Linalool, camphor, nerolidol, and safrole were provided by Guangzhou Guangyi Spice Co., Ltd. Nutrient agar and buffered peptone were purchased from Guangdong Huankai Microbial Science and Technology Co., Ltd. All other reagents were of analytical grade.

### 2.2 Experimental microbe

*Escherichia coli* ATCC 25922 was provided by Inspection and Quarantine Technology Center of Guangdong Entry‐Exit Inspection and Quarantine Bureau. The stock culture of *E. coli* was maintained on nutrient agar slopes at 4°C and subcultured every other week.

Bacterial suspension preparation: Strain was activated in nutrient broth for 24 hr and streaked onto nutrient agar plate. Single colony was picked to make bacterial suspension at the concentration of 1.5 × 10^8^ CFU/ml (0.5 McFarland units).

### 2.3 Treatment of *Escherichia.coli* with vapor‐phase EOL

*Escherichia.coli* was treated with EOL as described previously by Lopez et al (Goñi et al., 2009). Briefly, 100 μl bacterial suspension (0.5 McFarland units) was evenly spread on nutrient agar plates. Appropriate amount of EOL was added to the center of the cover of Petri dish. Petri dishes were cultured invertedly at 37°C for 24 hr after sealing with parafilm and forming gas atmosphere of essential oils by heating covers with water bath.

### 2.4 Determination of MIC and MBC of vapor‐phase EOL

MIC was measured by determining the lowest concentration of EOL that can inhibit the visible bacterial growth, while MBC was measured by determining the lowest concentration of EOL that can kill bacteria. EOL‐treated plates without bacterial growth were exchanged with a different cover to remove the essential oil vapor and continued to culture at 37°C for 24 hr. The lowest concentration of EOL corresponding to the plates with bacterial growth represents MIC, and the lowest concentration without bacterial growth represents MBC.

### 2.5 Crystal violet assay

Crystal violet assay was performed as described previously (Devi et al., 2010). Briefly, bacteria were collected from plates and dispersed into 8 ml of crystal violet (10 μg/ml in PBS) solution. After incubation at 37°C for 10 min, bacterial suspension was centrifuged at 4°C for 15 min (7,104 g). Optical density at 590 nm (OD_590_) was measured for the supernatant using a spectrophotometer. The absorption rate of the crystal violet was calculated by the following equation: Absorption rate = ((OD_590_ of crystal violet solution − OD_590_ of supernatant)/OD_590_ of crystal violet solution) × 100%.

### 2.6 Conductivity test

The Bacteria was treated by linaloe wood oil at various concentrations (100–800 μl/L) for different time (0.5–3.5 hr) in the culture plate. The treated bacteria were collected by washing the plate using 5 ml sterile water. After the suspension adjusted to 7.0 McFarland units, the conductivity was measured using turbidimeter.

### 2.7 Electronic microscopic analysis

Bacteria were collected from the plate, fixed, and observed under a scanning electron microscope (*SEM*) and a transmission electron microscope (TEM) as described previously(Chen et al., 2015, 2016; Tyagi & Malik, 2010).

### 2.8 Fourier transform infrared spectroscopy

Bacteria were collected from the plates using 10 ml of PBS (0.05 mol/L) and proceeded with infrared spectroscopic analysis as described previously (Al‐Qadiri, Al‐Alami, Al‐Holy, & Rasco, 2008; Chen, Zhang, Huang, Fu, & Liu, 2017). Characteristic spectrum of amide I (1600–1700 cm^−1^) was analyzed by Peak Fit v4112 software. The baseline was corrected, and then, deconvolution was performed using Gaussian. Subsequently, the second derivative was performed for the curve fitting and minimization of the residuals. The content of the secondary structure was calculated according to the peak area.

### 2.9 Fluorescence spectroscopy

Bacteria were collected from the plates using 10 ml of PBS (0.05 mol/L) and proceeded with fluorescence spectroscopic analysis as described previously (Pinotsi et al., 2016; Wachsmuth et al., 2015).

### 2.10 GC/MS analysis

Gas chromatograph was performed using Agilent HP6890 equipped with DB‐17MS silica‐capillary column (30 m × 0.25 mm, 0.25 μm film thickness, Agilent). The injector temperature was 250°C, the carrier gas was helium of high purity, carrier gas flow rate was 1.0 ml/min, column temperature was increased from 50 to 280°C, the solvent was delayed for 2.15 min, the injection volume was 0.2 μl, and the split ratio was 1:50. Agilent 5,973 was used for MS analysis. The MS interface temperature was 250°C, ionization mode was EI, the electron energy was 70 eV, the ion source temperature was 230°C, and the scanning range was 20–550 m/z. Solid‐phase microextraction (SPME) was performed with polydimethylsiloxane (PMDS, 100 μm) fibers for 24 hr at room temperature. Chemical composition of the essential oils was identified by NBS75K and WILEY275 on mass spectrometry data.

### 2.11 Quantum chemical analysis

All quantum chemical parameters were calculated by Material Studio 5.5. Density functional theory (DFT) (B3LYP/6‐31G(d)) was used as the model and calculated by high‐performance computer cluster platform (HP DL5800) in Guangdong University of Technology.

## 3 RESULTS AND DISCUSSION

### 3.1 Comparison of the vapor‐phase antibacterial activity between EOL and other essential oils

MICs and MBCs for different essential oils were determined (Table 1). The results showed that antibacterial activity of EOL and tea tree oil was much higher than that of other essential oils. A large number of studies have shown that tea tree oil has antibacterial activity (Pérez‐Rosés, Risco, Vila, Peñalver, & Cañigueral, 2015), but little is known on the antibacterial activity of EOL.

**Table 1:** Comparison of the vapor‐phase antibacterial activity between EOL and other essential oils

Essential oils	MIC (μl/L)	MBC (μl/L)	Essential oils	MIC (μl/L)	MBC (μl/L)
EOL	200	200	Eucalyptus oil	500	1,000
Tea tree oil	200	200	Star‐anise oil	500	1,500
Orange oil	250	250	Clove oil	1,000	1,000
Cassia oil	500	500	Mentha arvensis oil	1,500	1,500
Marjoram oil	500	500	Litsea cubeba oil	1,500	1,500
Rosemary oil	500	500	Clary sage oil	2,500	2,500

### 3.2 Effect of vapor‐phase EOL treatment on the permeability of *Escherichia.coli*

**Figure 1:**
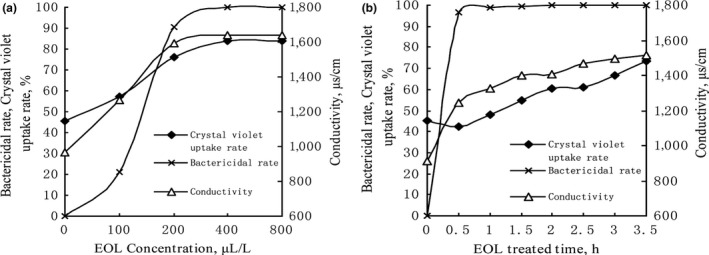
Effect different concentration and treatment time of vapor‐phase EOL on the permeability of *Escherichia.coli*

Bactericidal activity, absorption rate of crystal violet, and conductivity were increased with the increases of the vapor‐phase EOL concentration (Figure 1). However, when vapor‐phase EOL concentration was over 200 μl/L, bactericidal activity, absorption rate of crystal violet, and conductivity were not increased significantly (Figure 1). Bactericidal activity reached 100% after 30 min treatment and further extension of treatment did not enhance its bactericidal activity significantly. In contrast, absorption rate of crystal violet and conductivity were increased with the extension of the treatment. Therefore, we suspected that treatment with vapor‐phase EOL enhanced the permeability of *E. coli* cell membran*e*, leading to the leakage of intracellular substances (e.g., ions), and the elevation of conductivity. At the same time, the extracellular macromolecular substances also easily enter the bacteria, resulting in the increases of the absorption rate of the crystal violet. Similar results were observed when *Salmonella* were treated with clove oil (Devi et al., 2010).

### 3.3 Effect of vapor‐phase EOL treatment on the ultrastructure of *Escherichia.coli*

**Figure 2:**
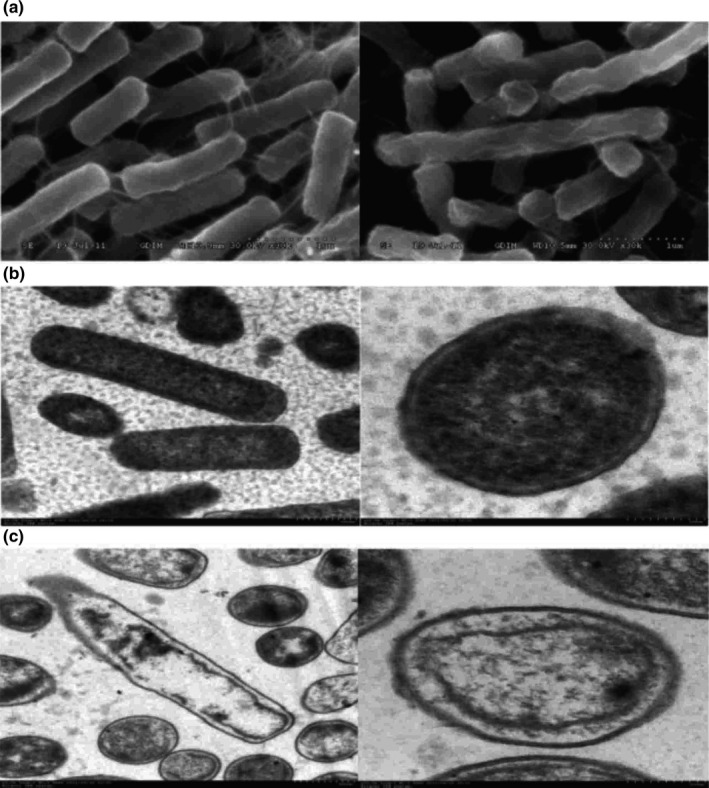
The morphology of *Escherichia.coli* observed by *SEM* and TEM

Normal *E. coli* has a typical short rod or cylindrical shape with full body and smooth surface (Figure 2A). After treatment with vapor‐phase EOL, bacterial surface became shrunken and shriveled. Further TEM analysis showed that normal *E. coli* had an integral cell wall and membrane structure with evenly distributed cytoplasm (Figure 2B). After treatment with vapor‐phase EOL, cell wall structure remained intact, but the cell membrane appeared partial rupture, leading to the leakage of the intracytoplasmic materials. In addition, cell membrane and cell wall were separated and the cytoplasm became unevenly distributed (Figure 2C). These results suggested that EOL treatment disrupted the cell membrane structure, leading to the increase of cell permeability, leakage of intracellular substances, and shrinkage of bacterial shape.

### 3.4 Effect of vapor‐phase EOL treatment on the tertiary structure of bacterial protein

**Figure 3:**
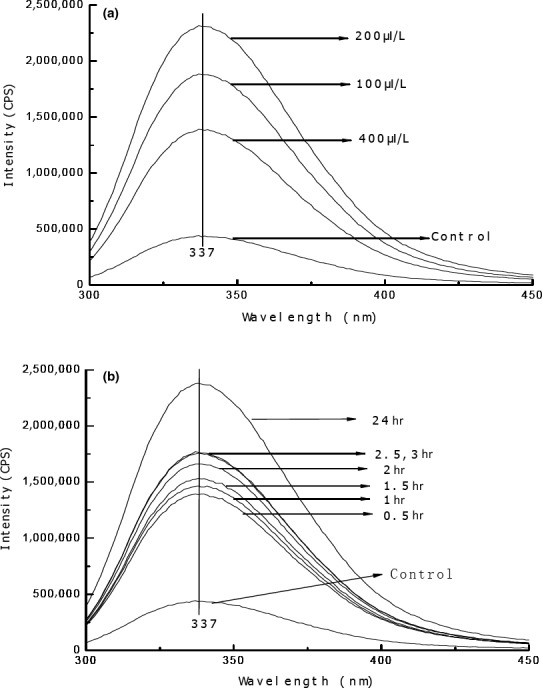
Effect of vapor‐phase EOL on the protein fluorescent intensity

The experiments described above demonstrated that vapor‐phase EOL treatment destroyed the structure of the cell membrane of *E. coli*, leading to the increase of cell permeability. Protein, fatty acids, and polysaccharides are the macromolecules of cell membrane. In *E. coli*, protein represents half of cell membrane. Therefore, we determined the effect of vapor‐phase EOL treatment on the tertiary structure of bacterial protein. The results showed that EOL treatment did not alter the maximum fluorescent emission peak, but the intensity was significantly changed (Figure 3). Fluorescent intensity was increased with the increases of EOL concentration from 0 to 200 μl/L (Figure 3A) or extension of treatment time at the EOL concentration of 200 μl/L (Figure 3B). However, fluorescent intensity at the EOL concentration of 400 μl/L was lower than that at 200 μl/L (Figure 3A). The alteration of *E. coli* protein fluorescent intensity indicated that EOL treatment altered the tertiary structure of bacterial protein. The increase of concentration or extension of EOL treatment facilitated fully spreading of protein molecules and exposure of more chromophores, leading to the increase of fluorescence intensity. However, when EOL concentration is too high (>200 μl/L), spreaded protein molecules become curled and folded, which results in the internalization of the exposed chromophores.

### 3.5 Effect of vapor‐phase EOL treatment on the secondary structure of bacterial protein

**Figure 4:**
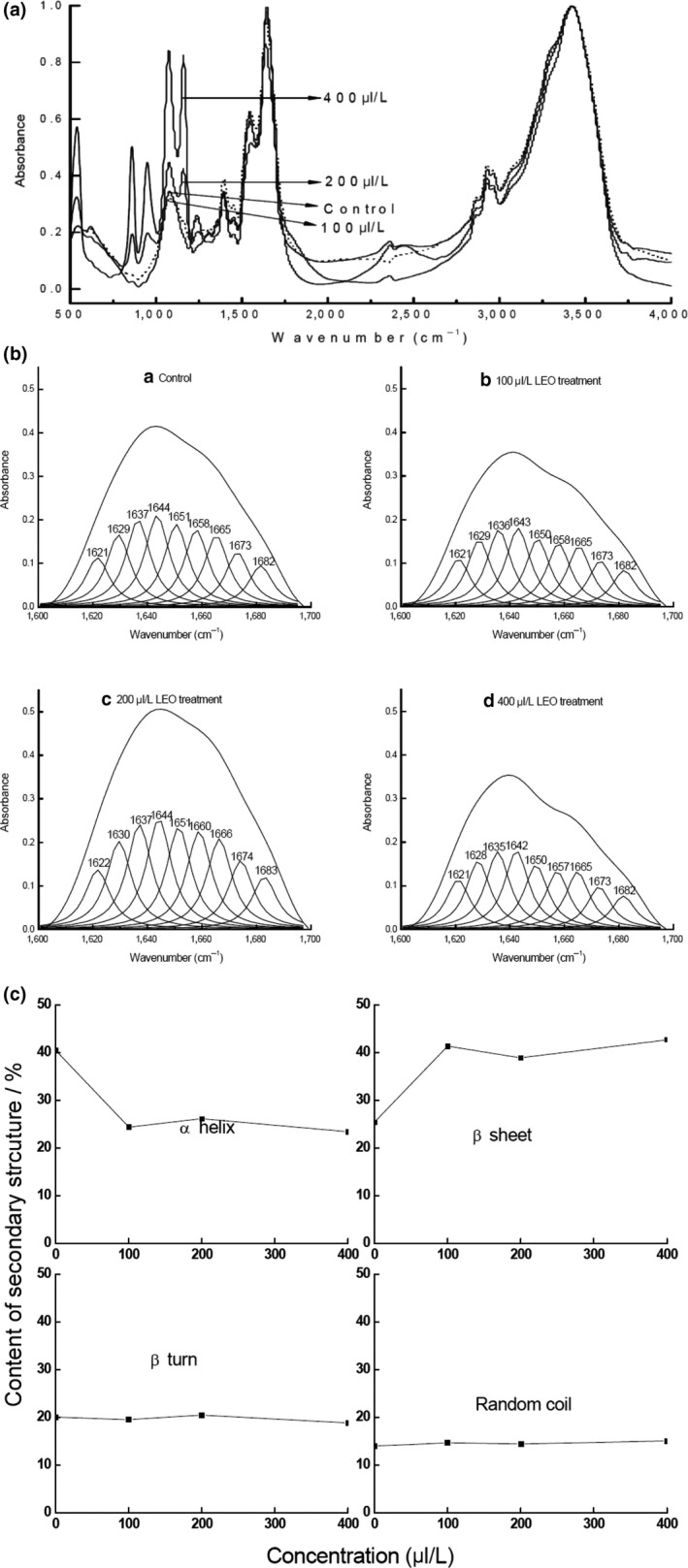
Effect of vapor‐phase EOL treatment on the secondary structure of bacterial protein

The amide I (1600–1700 cm^−1^ region) in the infrared spectrum is mainly the absorption of stretch vibration of C = O bond in the amino acids and it reflects α‐helix, β‐sheet, β‐turn, and random coil conformation (Figure 4A). To gain more information, we utilized second derivative and deconvolution to analyze the amide I region and obtained 9 subpeaks after curve fitting (Figure 4B). Based on the literature (Güler, Vorob'ev, Vogel, & Mäntele, 2016; McClements & Decker, 2000; Rolere, Liengprayoon, Vaysse, Sainte‐Beuve, & Bonfils, 2015), the peaks at 1615–1637 cm^−1^ and 1682–1700 cm^−1^ are β‐sheet, the peak at 1646–1664 cm^−1^ is α‐helix, the peak at 1664–1681 cm^−1^ is random coil, and the peak at 1664–1681 cm^−1^ is β‐turn. The effect of EOL treatment on each secondary structure was shown in Figure 4C. The results showed that vapor‐phase EOL treatment greatly reduced the content of α‐helix and increased the content of β‐sheet. These results indicate that EOL alters the intramolecular hydrogen bond arrangement and changes the α‐helix of peptide into linear structure, which leads to the alteration of bacterial protein secondary structure. However, β‐turn and random coil content was not significantly affected by EOL treatment (Figure 4C).

### 3.6 Antibacterial activity of components in the vapor‐phase EOL

**Figure 5:**
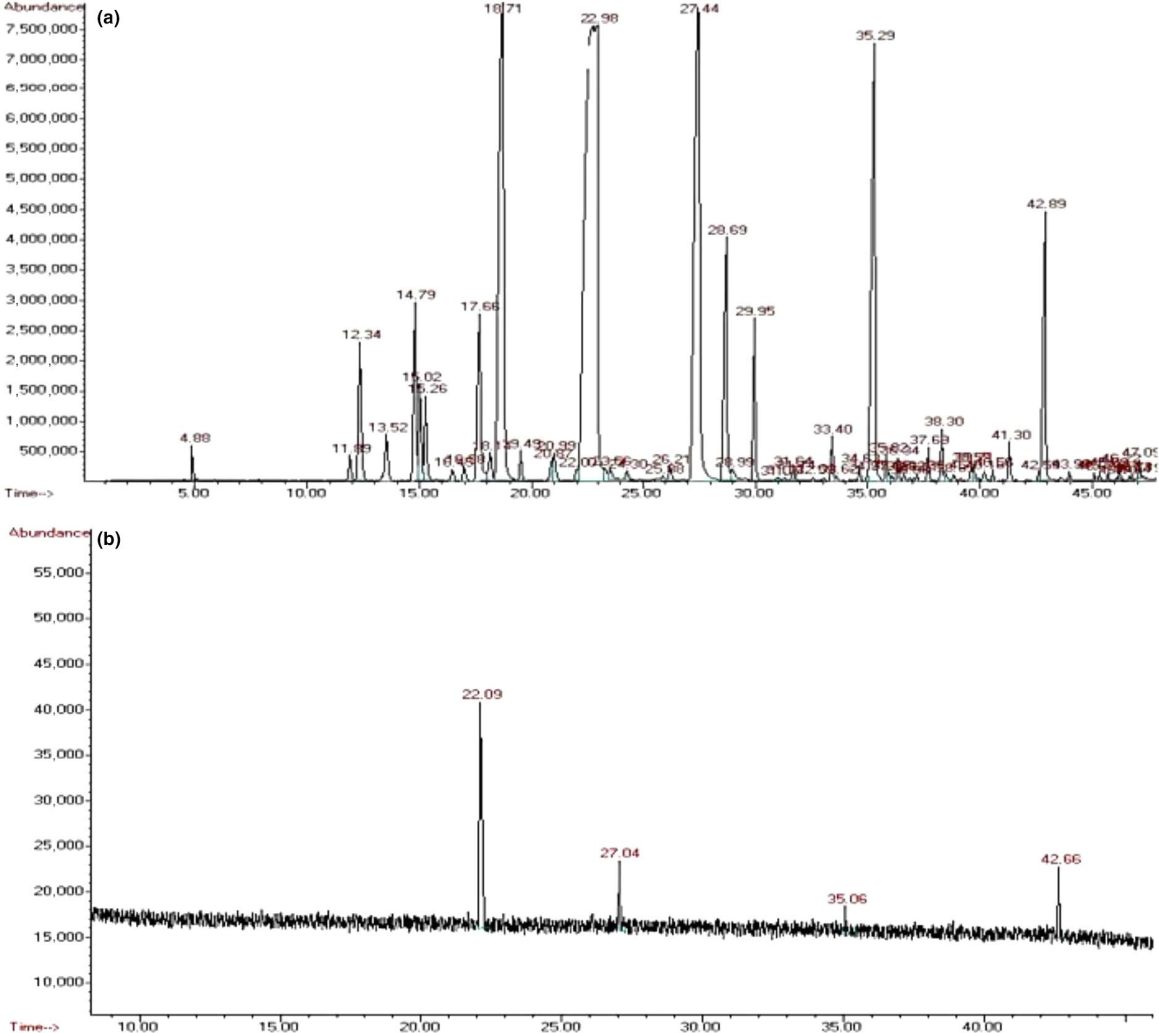
GC/MS analysis of EOL and its vapor phase

GC/MS analysis identified 45 main chemical components accounting for 99.53% of EOL (Figure 5A). However, only four components (linalool, camphor, nerolidol, and safrole) were identified in the vapor‐phase EOL (Figure 5B and Table 2). MICs and MBCs for components of vapor‐phase EOL were shown in Table 3. MIC and MBC of vapor‐phase EOL were similar to those of linalool, while there was no antibacterial activity for camphor, nerolidol, and safrole at the highest experimental concentration (3,000 μl/L). These results suggest that the antibacterial activity of vapor‐phase EOL is mainly derived from linalool.

**Table 2:** The main components of the vapor‐phase EOL

Number	Retention time	Components	Percentage of the vapor phase	Percentage of EOL
1	22.093	Linalool	69.94	35.17
2	27.045	Camphor	10.90	15.36
3	35.060	Safrole	8.24	8.34
4	42.658	Nerolidol	10.92	3.54

**Table 3:** Antibacterial activity of components of vapor‐phase EOL

	EOL	Linalool	Camphor	Nerolidol	Safrole
MIC (μl/L)	250	200	>3,000	>3,000	>3,000
MBC (μl/L)	250	250	>3,000	>3,000	>3,000

### 3.7 Quantum chemical analysis of linalool

Studies have shown that the antibacterial activity of a substance is closely correlated to its energy of the highest occupied molecular orbit (E_HOMO_) and the energy of the lowest unoccupied molecular orbit (E_LUMO_) (18). E_HOMO_ reflects the ability of the molecule to donate electrons. Higher E_HOMO_ indicates unstable electron in that orbit. E_LUMO_ reflects the ability of the molecule to receive electrons. Higher E_LUMO_ indicates more energy reduction was due to the electron entering that orbit. Electron transfer occurs when antibacterial substances act on the microbes, thus affecting the normal physiological function. The quantum chemical parameters of linalool were shown in Table 4. The results showed that linalool had higher E_HOMO_ and lower E_LUMO_, suggesting that it had a strong electron‐donating and a weak electron‐receiving ability. Therefore, the main electron transfer occurred during antibacterial action of linalool is the donation of electron.

**Table 4:** Quantum chemical parameters of linalool

E_HOMO_ (eV)	E_LUMO_ (eV)	E_HOMO_‐E_LUMO_ (eV)	E_total_ (Ha)	μ_d_ (au)
−5.306	−0.280	−5.026	−466.61	0.71884

**Figure 6:**
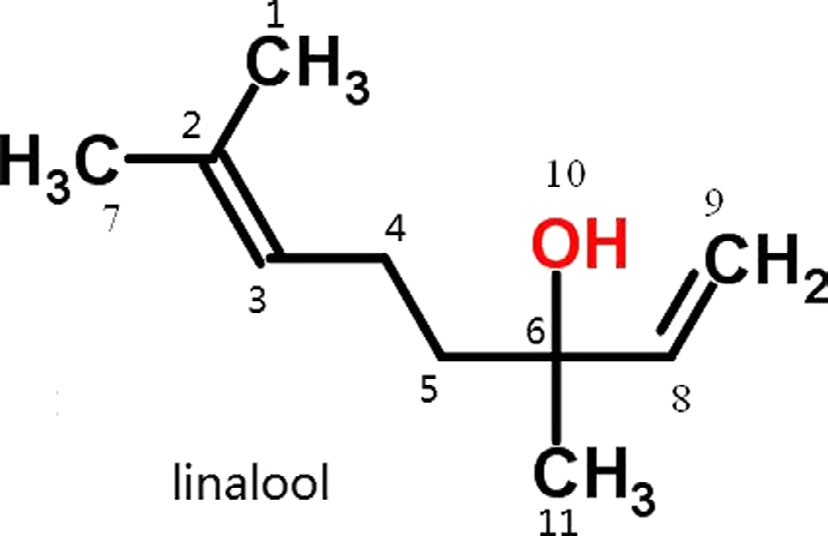
Atom numbering of linalool

Linalool atom numbering was shown in Figure 6, and the electron density of each atom was shown in Table 5. The results showed that the linalool atom with higher electron density was O_10_. Qin et al., showed that a distance of about 0.25 nm between electron acceptor center and electronic supply center is necessary for the reactive center of the antibacterial activity (Chrysargyris, Xylia, Botsaris, & Tzortzakis, 2017; Moghimi, Ghaderi, Rafati, Aliahmadi, & McClements, 2016; Zhang, Liu, Wang, Jiang, & Quek, 2016). The electron transfer usually occurs first in the atoms with higher electron density. Thus, O_10_ is the easiest atom to transfer electrons and is the electron‐donating center during linalool antibacterial process.

**Table 5:** Electron density of each atom in linalool

Atom	Electron density/au	Atom	Electron density/au	Atom	Electron density/au
C_1_	0.326	C_5_	0.193	C_9_	−0.088
C_2_	−0.022	C_6_	−0.114	C_10_	−0.526
C_3_	−0.067	C_7_	−0.080	C_11_	−0.034
C_4_	−0.028	C_8_	0.068		

Fukui function of linalool was calculated by Mulliken algorithm and the electrophilicity, nucleophilicity, and free radical affinity were obtained (Table 6). The results showed that hydroxy group with O_10_ atom has a strong electrophilicity and free radical affinity, which is very important for linalool to disarrange the intramolecular hydrogen bond arrangement of protein during antibacterial process. These results further confirmed that O_10_ is the reactive center of antibacterial activity of linalool.

**Table 6:** Electrophilicity, nucleophilicity and free radical affinity of each atom in linalool

Atoms in linalool	Electrophilicity	Nucleophilicity	Free radical affinity
C_1_	−0.034	−0.041	−0.037
C_2_	−0.011	−0.027	−0.019
C_3_	−0.028	−0.029	−0.028
C_4_	0.047	0.052	0.049
C_5_	0.063	0.062	0.062
C_6_	−0.027	−0.014	−0.021
C_7_	−0.031	−0.023	−0.027
C_8_	−0.017	0.098	0.041
C_9_	0.031	0.104	0.067
C_10_	0.155	0.034	0.094
C_11_	−0.020	−0.024	−0.022

## 4 CONCLUSION

EOL had a significant vapor‐phase antibacterial activity with an MIC and MBC of 200 μL/L. Vapor‐phase EOL treatment led to the bacterial cell membrane rupture, increased permeability of the bacterial cell, leakage of intracellular substance, and alteration of the protein structure, thereby affecting the normal growth and the physiological metabolism of the *E. coli*. The main antibacterial component of the vapor‐phase EOL is linalool, and the antibacterial reactive center of linalool is oxygen atom O_10_ which transfers electrons during antibacterial action of linalool by the donation of electrons.

## CONFLICT OF INTEREST

Authors declare they have no conflicts of interest.

## ETHICAL APPROVAL

This article does not contain any studies with human or animal subjects performed by any of the authors.
